# Do preheated composite resins provide better cementation results for indirect restorations? A Systematic Review

**DOI:** 10.4317/jced.62356

**Published:** 2025-01-01

**Authors:** João Paulo do Vale Souza, Lucas Tavares Piacenza, José Vitor Quinelli Mazaro, André Luíz de Melo Moreno, Nathaly Vilene de Araújo Moreno, Daniela Micheline dos Santos, Marcelo Coelho Goiato

**Affiliations:** 1 Department of Dental Materials and Prosthesis, Faculty of Dentistry of Araçatuba, São Paulo State University (UNESP); 2 Courses for Dentistry (Instituto de Excelência em Ensino e Pesquisa), Manaus, Amazonas, Brazil

## Abstract

**Background:**

The article addresses the use of preheated composite resins as cementation agents in indirect restorations, highlighting the importance of these materials in the adhesion between the restoration and the dental substrate. Cementation agents are crucial for the overall strength of restorations, and research indicates that resins with higher inorganic filler content may enhance the strength of ceramic restorations.

**Material and Methods:**

The study was conducted as a systematic review, following PRISMA guidelines, and analyzed bond strength, physicochemical properties, and color variations. After a search in various databases, nine studies were included in the analysis.

**Results:**

The results suggest that while preheated composites may have some advantages, their mechanical properties and the thickness of the cementation layer are not superior to those of traditional resin cements.

**Conclusions:**

The article concludes that the adoption of these composites as cementation agents results in modest improvements, and caution is needed when considering their clinical application.

** Key words:**Composite Resins, Cementation, Dental Prosthesis.

## Introduction

Cementation agents are considered the most susceptible part of a restoration, as they rely on adhesion between the restoration and the substrate, thereby affecting the overall strength of the system (1). Studies indicate that a higher content of inorganic fillers in resin-based cementation agents can enhance the strength of thinner ceramic restorations (2,3). However, resin cements used as cementation agents (4,5) generally have a lower concentration of inorganic fillers compared to other resin-based materials, such as conventional composite resins (RCs) (6). For this reason, the use of composite resins as cementation agents has been suggested (4,7), as they offer benefits such as a wide range of shades and superior mechanical performance due to their higher filler content (4,8).

Cementation agents play a crucial role in enhancing the strength of indirect restorations by infiltrating the porosities of the internal ceramic surface created by acid etching (9), and they also contribute to sealing cracks on the surface(10). The high viscosity of composite resins (RC) (11) may not only increase the thickness of the cement layer but also compromise the adaptation of the restoration (12). The polymerization of preheated RCs used as cementation agents can induce significant stresses in the cement layer, favoring the formation of cracks in thinner ceramic restorations (13). However, studies show that preheating the material reduces its viscosity, thereby facilitating its penetration into the previously etched ceramic (3,14). Although there is still no consensus on the ideal thickness of the cement layer (15), most experts recommend that this thickness should be less than 120 µm (16,17).

The color variation of composite resin restorations, influenced by the content of inorganic fillers, has also been investigated (18). Considering that color change can be affected by various factors (19) and that achieving restorations that mimic natural teeth remains one of the main challenges in restorative dentistry, color stability emerges as a relevant topic in aesthetic dentistry. A previous systematic review recommended the analysis of the physical properties of preheated materials, including RCs (20). However, the impact of using preheated RCs as cementation agents on the performance of indirect restorations is a significant topic in clinical practice that has yet to be comprehensively explored in the literature.

Thus, this systematic review aimed to investigate whether the use of preheated composite resins as cementation agents can enhance the mechanical properties, physical-chemical performance, and color of indirect restorations.

## Material and Methods

This systematic review was conducted in accordance with the guidelines of the Cochrane Handbook and followed the four-phase flow diagram outlined in the PRISMA (Preferred Reporting Items for Systematic Reviews and Meta-Analyses) guidelines. The study protocol was registered with the International Prospective Register of Systematic Reviews (PROSPERO) (CRD42020140696), and the research question was structured using the PICO format, which includes: population (teeth undergoing indirect restorations); intervention and comparison (the use of preheated composite resins versus resin cements as cementation agents); and outcomes, including mechanical properties (such as bond strength, shear bond strength, and flexural strength), physical-chemical performance (such as cement line thickness and color change). The formulated research question was: “Do preheated composite resins provide better cementation results for indirect restorations?” 

-Search Strategy and Selection Process

A search strategy was developed encompassing the databases Medline/PubMed, Scopus, and Web of Science, as well as gray literature (OpenGrey, ProQuest, and the CAPES Theses and Dissertations Catalog), with the final search conducted in August 2024. No restrictions were placed on language or publication dates.Studies were included if they compared at least one preheated composite resin as a cementation agent to resin cements. Studies that analyzed preheated composite resins but did not assess the mechanical properties of the bonded restorations were excluded. Literature reviews, case series, and studies using experimental materials or not adhering to manufacturer recommendations were also excluded.

Studies meeting the eligibility criteria were initially identified by two independent reviewers who analyzed the titles. Subsequently, the abstracts were assessed, and the full texts were reviewed in cases where relevant information was missing from the abstracts. Only articles that met the eligibility criteria were considered for data extraction. Additionally, manual searches were conducted on the references of all selected articles, and any discrepancies regarding the inclusion of studies were discussed with a third reviewer for resolution.

-Assessment of Risk of Bias

The risk of bias for each study was independently assessed by two reviewers, JPVS and 

TP, based on criteria adapted from a previous systematic review on *in vitro* studies (21). The aspects evaluated included: randomization of samples, calculations to determine sample size, blinding of the operator during tests, sufficient detailed information to allow replication, and other potential sources of bias. Each of these aspects was categorized as high risk (when the criterion was not met and clearly stated), low risk (when the criterion was met and clearly reported), or uncertain risk (when there was no information regarding the criterion’s fulfillment). This analysis was conducted using RevMan (Review Manager Software®, version 5.2, Nordic Cochrane Centre, Cochrane Collaboration, Copenhagen, Denmark).

## Results

-Description of Studies

**Figure 1:**
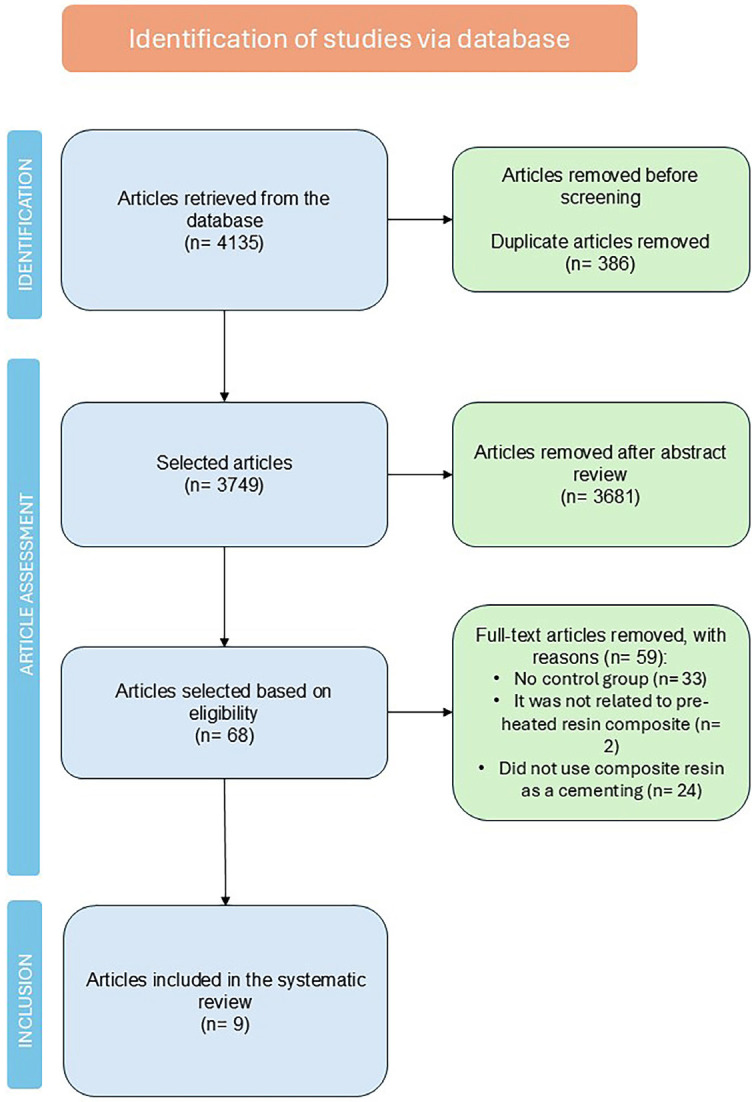
Search strategy information for the selection of articles for this review.

The search strategy yielded 4,135 articles (Fig. 1), and 3,749 publications were analyzed after duplicate removal. No documents were identified through manual search, and 3,681 studies were excluded for not meeting the eligibility criteria. Consequently, 68 articles were selected for full reading. Fifty-nine studies were excluded for the reasons presented in Fig. 1, resulting in 9 studies included in this review. No clinical studies were identified. Five studies were included in the meta-analysis, while 4 studies were analyzed qualitatively only, as they assessed properties of materials not investigated in the other included studies. The materials and main outcomes of each included study are detailed in  Table 1 and  Table 2.

-Qualitative and Descriptive Analyses

The evaluated properties, including shear bond strength (22), color change (18), and cement line thickness (23,24), were investigated through qualitative analysis (as presented in  Table 1 and  Table 2). Bond strength (23,25,26) and film thickness (3,22-24) were examined both qualitatively and quantitatively. The selected studies assessed the properties of the cementing agent in relation to the overall performance of the restorations.

Three investigations focused on bond strength. One study (25) compared a resin composite (Filtek Z250®, 3M ESPE, MN, USA) with three resin cements: a dual-cure cement (RelyX Unicem®, 3M ESPE, MN, USA), a conventional dual-cure cement (RelyX ARC®, 3M ESPE, MN, USA), and another dual-cure cement (Clearfill Esthetic®, Kuraray, Tokyo, Japan). Another study (26) analyzed two resin composites (Filtek Z250®, 3M ESPE, MN, USA; and Venus®, Heraeus Kulzer, Weihrheim, Germany), both in preheated and non-preheated conditions, compared to a conventional dual-cure resin cement (RelyX ARC®, 3M ESPE, MN, USA). The third study (24) evaluated the resin cement RelyX U200® (3M ESPE, MN, USA) in comparison with the nanohybrid ENA HRi® (Synca, Le Gardeur, CA, USA). In the first two studies (25,26), the preheated resin composite demonstrated superior bond strength. In contrast, the third study (24) indicated that the use of resin cement resulted in greater bond strength.

Regarding shear bond strength (22), the cementing agent Panavia V5® (Kuraray, Tokyo, Japan), both with and without immediate dentin sealing, exhibited shear bond strength values superior to those of the preheated resin composite (Protect Liner F®, Kuraray, Tokyo, Japan). One study compared different resin cements with two composites (Empress Direct®, Ivoclar Vivadent, Schaan, Liechtenstein; and Filtek Supreme Ultra Universal®, 3M ESPE, MN, USA), used with and without preheating. Preheating did not show a significant effect on volumetric contraction; however, the composites, regardless of preheating condition, exhibited considerably greater volumetric contraction compared to the tested resin cements (23). Furthermore, all studies that assessed film thickness (3,23,25) observed an increase in film thickness when employing preheated resin composites as cementing agents, compared to resin cements.

Two studies investigated color change (18). In one of these studies, three resin composites (Filtek Z100®, 3M ESPE, MN, USA; Herculite Classic®, USA; and Durafill®, Heraeus Kulzer, Weihrheim, Germany) were compared to a conventional light-curing resin cement (Allcem Veneer®, FGM, São Paulo, Brazil), both with and without preheating. Overall, the color variations remained within the acceptability limits ΔE (ΔEab= 2.66 and ΔE00= 1.77) for all tested groups after one year of storage in distilled water.

Four distinct studies(24) evaluated the thickness of the cement line. A preheated resin composite (ENA HRi®, Synca, Le Gardeur, CA, USA) was compared to a self-adhesive cement (U200®, 3M ESPE, MN, USA), and no significant difference in the level of microleakage was observed between the preheated composite and the resin cement. Analysis conducted through scanning electron microscopy on longitudinal sections of indirect composite restorations cemented with resin revealed the presence of gaps at the interface between the tooth and the restoration. Additionally, it was found that the interface thickness was narrower on the walls and at the bottom of the cavity for restorations cemented with the preheated resin composite.

## Discussion

This systematic review represents the first initiative aimed at consolidating *in vitro* evidence related to the mechanical properties, physicochemical performance, and color variations in indirect restorations cemented with conventional preheated resin composites, highlighting that the type of cementing agent has a substantial impact on the analyzed outcomes.

The evaluation of bond strengths in a global analysis did not reveal significant differences. In fact, subgroup analysis comparing preheated resin composites to different resin cements indicated that the preheated resin composites exhibited mechanical properties comparable to those of self-adhesive resin cements, but inferior to those of conventional cementing agents. The equivalence in bond strength observed between the preheated resin composites and the self-adhesive cements can be attributed to the presence of acidic monomers (such as 10 MDP) in the formulation of the self-adhesive cement, which act as diluents, reducing the viscosity of the material and increasing its wettability, as well as promoting interaction with dental tissues (27). The main variations in the composition of resin cements and resin composites are generally associated with the amount of inorganic filler particles and the type of organic matrix, factors that influence the mechanical properties of the composites. Compared to resin composites, resin cements tend to have a more organic matrix and a lower concentration of inorganic filler particles (28).

Regarding the film thickness, both the overall meta-analysis and subgroup analyses indicated that the preheated resin composites exhibited a film thickness considered clinically unacceptable (>120 µm). The increase in the proportion of organic matrix in resin cements favors more efficient flow, resulting in a lower film thickness than that observed in preheated resin composites.

A previous study highlighted that the thickness of the films formed by preheated resin composites was 4.5 times greater than that of the films generated by dual-cure resin cements when the same adhesive system and load were applied, corroborating the findings of this meta-analysis. It is recommended that cementing agents should be able to form a film thickness that does not exceed 120 µm. Recently, a single study indicated that the Filtek Z100® resin preheated to 69°C, using the Hotset® system, generated a film thickness that is considered clinically acceptable, around 100 µm. Films that exceed the recommended limits may lead to increased exposure of the cementing agent to the oral environment, resulting in inadequate marginal adaptation and an increase in microleakage over extended follow-up periods of indirect restorations. A recent study indicated that the variation in film thickness may be attributed to the distinct preheating behavior of the various resin cement formulations (15).

However, there is moderate evidence that microleakage tests using dye penetration do not show correlation with any of the evaluated clinical parameters, such as postoperative hypersensitivity, retention, and marginal stains (29).

It is imperative to highlight some limitations of this review. Only one of the analyzed studies implemented blinding of the operator responsible for the tests. Furthermore, most *in vitro* studies rarely evaluate dental materials, as noted in other reviews (21,29). This issue is particularly relevant, as an experienced operator can easily distinguish between different types of samples, which may influence the interpretation of the results.

Additionally, none of the considered studies calculated the sample size. The researchers opted to use a sample size similar to that of previous investigations or suggested by the standards (22). Studies that did not follow the manufacturer’s recommendations were excluded from this systematic review to minimize bias, resulting in a low risk of bias for most of the evaluated parameters.

## Conclusions

Considering the limitations identified in this study, this review suggests that the use of preheated resin cements as cementing agents results in only modest improvements in the mechanical properties of indirect restorations. Furthermore, it was observed that the physicochemical characteristics of these cements are either similar to or inferior to those of conventional resin cements, including a film thickness deemed clinically unacceptable.

## Figures and Tables

**Table 1:** Information on each included study.

Article	Substrate	Restorative material	Evalueted properties
Alvarado et al., 2020	Human third molars	Prefabricated composite resin restorations	Marginal seal Microleakage Microtensile bond strength
Coelho et al., 2019	Composite resin	Overlaid blocks of composite resin and lithium disilicate ceramic	Film thickness Biaxial flexural strength
Mounajjed et al., 2018	Human third molars	Lithium disilicate ceramic crowns	Cement line thickness
Goulart et al., 2018	Human third molars	Indirect composite resin restorations	Microtensile bond strength
Gugelmin et al., 2020	Incisivos bovinos	Lithium disilicate ceramic veneers	Color change
Reboul et al., 2018	Human third molars	Lithium disilicate blocks	Shear strength
Sampaio et al., 2016	Inicisivos centrais de plástico	Plastic veneers	Cement line thickness Polymerization shrinkage
Sartori et al., 2016	Human third molars	Leucite-reinforced glass-ceramic plates	Microtensile strength Cement line thickness
Almeida et al., 2015	Discos de esmalte dentário bovinos	Ceramic discs	Color change

**Table 2:** Description of the included studies and their quantitative and qualitative results.

Results	Bonding Agents	Types of Composite Resins	Heating Temperature	N	Main Results	Statistical Analysis
Microshear Bond Strength						
Sarrioni et al., 2016	Filtek Z350	Microhybrid	68ºC	3	Filtek Z350 showed statistically higher bond strength compared to the other groups.	35.58 (0.3) MPa*
	RelyX U200	Dual-cure self-adhesive cement	-	3		19.02 (1.8) MPa
	Clearfil Esthetic	Photopolymerizable resin cement	-	3		10.25 (1.19) MPa
	RelyX ARC	Dual-cure resin cement	-	3		18.13 (7.9) MPa
Goulart et al., 2018	Filtek Z350	Microhybrid	22ºC	5	In cementing 2mm restorations, only pre-heated or room temperature Filtek Z350 exhibited significantly higher bond strength than RelyX ARC.	36.59 (3.8) MPa*
	Venus	Submicron hybrid resin	22ºC	5		29.11 (0.3) MPa*
	Filtek Z350	Microhybrid	64ºC	5		31.12 (1.5) MPa*
	RelyX ARC	Dual-cure resin cement	-	5		27.78 (1.0) MPa
	Venus	Submicron hybrid resin	64ºC	5		25.42 (0.2) MPa*
Alvatado et al., 2020	ENAHR	Nano-hybrid resin	59ºC	15	The resin cement exhibited lower microshear bond strength compared to the composite resin.	14.42 (4.6) MPa*
	RelyX U200	Dual-cure self-adhesive resin cement	-	15		27.62 (8.1) MPa
Cementation Film Thickness						
Sarrioni et al., 2016	Filtek Z350	Microhybrid	68ºC	3	Pre-heated composite resins showed greater cementation line thickness.	443.41 (3.0) µm*
	RelyX U200	Dual-cure self-adhesive cement	-	3		168.13 (1.0) µm
	Clearfil Esthetic	Photopolymerizable resin cement	-	3		97.13 (1.0) µm
Sampaio et al., 2016	Filtek Supreme Ultra Universal	Nanoparticulate	68ºC	6	Pre-heated and non-pre-heated composite resins exhibited greater cementation line thickness compared to resin cements.	320 (30) µm*
	IPS Empress Direct	Nano-hybrid	-	6		310 (40) µm*
	IPS Empress Direct	Nano-hybrid	68ºC	6		310 (40) µm*
	RelyX Veneer	Photopolymerizable resin cement	-	6		210 (41) µm
Color Change						
Guguini et al., 2020	Alcem Veneer	Photopolymerizable resin cement	-	10	All resin cements and pre-heated or non-pre-heated composite resins exhibited similar color change, maintaining ΔE00 values below the acceptable limit.	ΔE00 = 2.0 (0.3)
	Filtek Z100	Microhybrid	-	10		ΔE00 = 2.1 (0.3)
	Herculite Classic	Microhybrid	-	10		ΔE00 = 2.4 (0.2)
	Durafill	Microparticulate resin	-	10		ΔE00 = 2.0 (0.3)
	Filtek Z350 XT	Nano-hybrid resin	60ºC	10		ΔE00 = 2.0 (0.3)
Almeida et al., 2015	RelyX ARC	Dual-cure resin cement	-	10		ΔE00 = 1.3 (1.6) *
	Filtek Z350 Flow	Nano-hybrid flowable resin	60ºC	10		ΔE00 = 2.0 (0.3)

## Data Availability

The datasets used and/or analyzed during the current study are available from the corresponding author.
